# Nipah virus on the radar: The choice to catch early or catch up later

**DOI:** 10.1016/j.nmni.2026.101732

**Published:** 2026-03-04

**Authors:** Hester Lacey, Shivani Jain, Aigars Reinis, Nityanand Jain

**Affiliations:** a University Hospitals Sussex NHS Foundation Trust, Eastern Rd, Brighton, BN2 5BE, United Kingdom; b Flinders University, Sturt Road, Bedford Park, South Australia, 5042, Australia; c Department of Oral and Maxillofacial Surgery, Genesis Institute of Dental Sciences and Research, Ferozepur-Moga Rd, Firozpur, Punjab, 152002, India; d Faculty of Medicine, Riga Stradinš University, Dzirciema Street 16, Riga, LV-1007, Latvia; e Joint Microbiology Laboratory, Pauls Stradinš Clinical University Hospital, Pilsonu Street 13, Riga, LV-1002, Latvia

**Keywords:** Nipah virus, Case definitions, Outbreak preparedness, One Health, Surveillance

In January 2026, India reported two laboratory-confirmed Nipah virus infections in West Bengal, the state's third recognised event after earlier outbreaks in 2001 and 2007. As notified to the World Health Organization (WHO), both cases occurred in the same private hospital in Barasat (North 24 Parganas, near Kolkata) and involved young healthcare workers with symptom onset reported in late December 2025. Extensive contact tracing and testing identified no additional infections [1,2]. In parallel, Bangladesh reported an unrelated fatal Nipah virus case in early February 2026, with contact investigations also yielding no new secondary cases [3]. Both incidents prompted a swift international response, including transient border screening and rapidly shifting risk communication [4]. Yet, we believe, such preventive measures are only as good as the signals they are built on. For Nipah, those signals are shaped by who is counted, how quickly, and by what definition. In this editorial, we argue that preparedness requires not only rapid public health action but also a common language for what counts as a “case”, because ambiguity can travel faster than the virus itself.

## 1 The pathogen and the syndrome

Nipah virus (*Henipavirus nipahense*) is a zoonotic, highly pathogenic, enveloped, negative-sense single-stranded RNA virus in the family *Paramyxoviridae* [5]. First identified during the outbreaks in 1998–1999, it is now recognised as a cause of severe disease in both humans and animals. At a molecular level, the virus uses two surface glycoproteins to attach to and enter host cells – the tetrameric receptor-binding protein (G/RBP) and the trimeric fusion (F) protein [6]. The Nipah virus G (or RBP) glycoprotein binds the highly conserved host cell-surface ligands ephrin-B2 and ephrin-B3 (EFNB2/3) as entry receptors, thereby triggering downstream activation of the F protein that drives membrane fusion [7]. Notably, it is this dual receptor binding that is thought to provide a mechanistic basis for the broad host range and tissue tropism typically observed in Nipah virus infections [6,7].

The virus is recognised by the WHO as a top priority disease for research and development, because of its high reported case-fatality, epidemic potential, and the absence of widely available, proven targeted therapeutics [8]. Clinically, incubation period typically lasts 3 to 14 days post-exposure. Early illness is often non-specific, with fever, headache, malaise, vomiting, and sometimes upper respiratory tract symptoms. In animals, disease is often predominantly respiratory while in humans, severe disease frequently presents with encephalopathy characterized by acute encephalitis with altered consciousness, seizures, and coma [9]. In fulminant cases, rapid multisystem deterioration has been described, and death may occur within 24 to 48 hours of symptom escalation. Management is primarily supportive, emphasizing early recognition, stabilization, and organ-supportive care (including ABCDE triage, airway/ventilatory support, seizure control, management of raised intracranial pressure where relevant) [10]. Such clinical heterogeneity in presentation, progression, and management is precisely why surveillance and clinical definitions matter in Nipah infections.

## 2 Reservoirs without borders

**Fig. 1:**
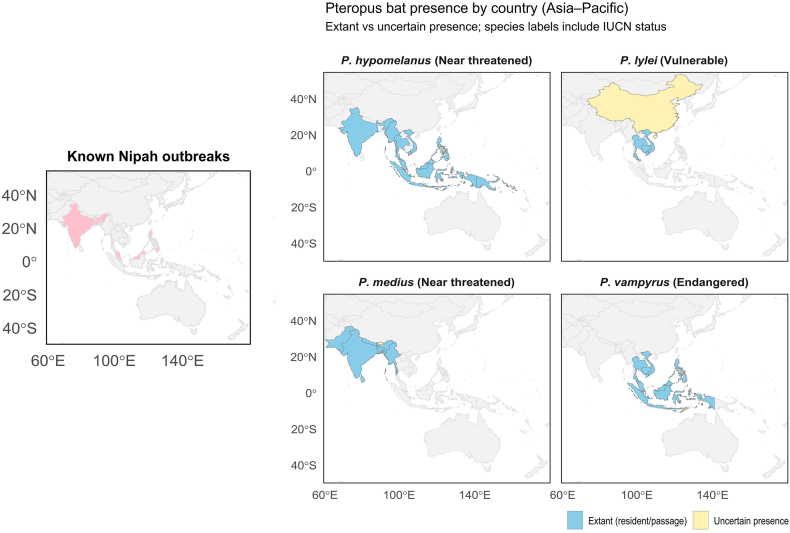
Geographic distribution of *Pteropus* fruit bats relevant to henipavirus ecology and locations of recognised Nipah outbreaks in the Asia–Pacific region. The four right-hand panels show country-level presence for different *Pteropus* species from the compiled dataset: *P. hypomelanus*, *P. lylei*, *P. medius*, and *P. vampyrus* (data sourced from the International Union for Conservation of Nature [IUCN] Red List of Threatened Species). IUCN Red List status shown in parentheses. Countries are shaded blue for extant occurrence (resident and/or passage) and pale yellow where presence is uncertain. The left-hand panel highlights countries with reported Nipah outbreaks (pastel red). Note that presence is displayed at country resolution and does not imply uniform distribution within national borders. The authors remain neutral with regard to jurisdictional claims and the depiction of territorial boundaries on maps.

The natural reservoirs of henipaviruses, including Hendra virus and Nipah virus, are pteropid fruit bats (*Pteropus* spp.), commonly known as flying foxes [11,12]. Human exposure to Nipah virus is believed to occur either via (i) direct contact with infected fruit bats or their secretions/excretions; (ii) ingestion of contaminated raw date palm sap; (iii) close contact with infected animals; or (iv) human-to-human transmission through close contact with an infected person's bodily fluids and respiratory secretions (including droplets). Nipah virus has been isolated and/or genetically characterised from several *Pteropus* species, including *P. vampyrus* (Malayan flying fox), *P. hypomelanus* (island flying fox), and *P. lylei* (Lyle's flying fox) in the Southeast Asian region, as well as *P. medius* (Indian flying fox) in India and Bangladesh (Fig. 1) [12]. Evidence of Nipah virus or Nipah-like henipaviruses has also been reported in bats from countries without recognised human outbreaks, including Cambodia, China, Indonesia, Madagascar, and Ghana [[12], [13], [14]]. It remains unclear whether this apparent lack of outbreak detection in other countries partly reflects surveillance intensity and/or the ecological and behavioural contexts that shape exposure risk, such as bat hunting, rapid urbanisation, climate change, and practices including the collection and consumption of raw date palm sap [13,15].

Furthermore, although recognised spillover has historically been geographically separated between Hendra virus (Australia) and Nipah virus (South and Southeast Asia), the ecological connectivity of bat populations remains relevant, as *Pteropus* species have been shown to undertake long-distance, migratory-like movements between roosts over large spatial scales [14]. Beyond humans, Nipah virus has been reported to infect a range of domestic animals (such as dogs, cats, pigs, and cows) and wildlife (such as wild boar, rodents, and birds); however, these species are generally considered incidental or dead-end hosts [11,16]. Pigs, however, are widely regarded as potential amplifying hosts [11], with some suggesting a potentially similar role for infected cows in the 2001 Bangladesh outbreak [17].

For surveillance purposes, the practical point is that animal events can be informative even when their role in transmission is uncertain. In the earliest Malaysian and Singaporean outbreaks, infection in farm pigs preceded recognition of human cases. The outbreak then subsided following movement restrictions: Singapore's ban on pig imports from Malaysia, and large-scale control measures in Malaysia, including the culling of over a million pigs [11]. Hence, animal morbidity/mortality signals and husbandry- or market-linked exposures can function as actionable early-warning context. Tiered case definitions and shared reporting fields should therefore capture exposure pathways explicitly (including differentiating bat contact, contaminated food, animal contact, healthcare exposure) and support linkage to relevant animal and environmental event signals where available.

## 3 Surveillance without a common language

Surveillance and epidemiological reporting for Nipah virus remain highly heterogeneous, reflecting, at least in part, absence of widely adopted WHO-standardized surveillance case definitions. In practice, although national guidance exists in several countries including Bangladesh [18], India [19], Pakistan [20], and South Africa [21], meaningful differences persist in how case definitions are operationalized. As summarized in Box 1, the Bangladeshi and Indian guidelines illustrate how variations in symptom requirements and epidemiologic links can potentially produce materially different case mixes. Furthermore, the Bangladeshi guidelines are notable for explicitly separating encephalitis-focused clinical criteria from structured definitions for clusters and linkages relevant to surveillance and outbreak investigation [18].

Box 1 **Case definitions as defined in the National Guidelines from Bangladesh and India.**

**Table:** 

Standard Case Definitions^1,2^	
**Confirmed case**	Any individual who has laboratory confirmation of Nipah virus infection either by • IgM antibody against Nipah virus identified in serum or cerebrospinal fluid (CSF) • Nipah virus RNA identified by PCR from respiratory secretions, urine, or CSF • Isolation of Nipah virus from respiratory secretions, urine or CSF *Note in Indian guidelines IgM antibody detection is not a criterion for confirmed case*
**Suspected case**	Any individual from a community affected by a Nipah virus disease outbreak who has • Fever with new onset of altered mental status or seizure and/or • Fever with headache and/or • Cough with shortness of breath *Note in Indian guidelines cough and dyspnoea must be accompanied with fever*
**Probable case**	Suspect case-patients who resided in the same village where confirmed case-patients were living during the outbreak period and who died before complete diagnostic specimens could be collected *Note in Indian guidelines, there is also an alternative criterion in addition with the above-mentioned criterion: Suspected case-patients came in direct contact with confirmed case-patients in a hospital setting during the outbreak period and who died before complete diagnostic specimens could be collected*
**Nipah Encephalitis Case Definitions ^1^**	
**Confirmed case**	A suspected or probable case with laboratory confirmation for Nipah infection either by • IgM antibody against Nipah virus by ELISA in serum or CSF • Nipah virus RNA identified by PCR from respiratory secretions, urine, or CSF
**Suspected case∗**	A person fulfilling both of the following criteria is defined as a suspected case 1 Features of acute encephalitis as demonstrated by • Acute onset of fever AND • Evidence of acute brain dysfunction as manifested by either (i) altered mental status OR (ii) new onset of seizure OR (iii) any other neurological deficit 2. Epidemiological linkage as documented in patient history with exposure to either • Drinking raw date palm sap OR • Occurring during Nipah season OR • Patient from Nipah endemic area
**Probable case∗**	A person with features of acute encephalitis and presents either • During a Nipah outbreak in the area OR • With history of contact with confirmed Nipah patient
**Cluster**	Two or more patients meeting the case definition who are within 21 days of each other and who live within 30 minutes walking distance of each other or have had contact with one another patient with similar illness.
**Other Definitions ^2^**	
**Contact case∗∗**	A close contact is defined as a patient or a person who encountered a Nipah case, either confirmed or probable cases, in at least one of the following ways • Was admitted simultaneously in a hospital ward/shared room with a suspected or confirmed case of Nipah • Has had direct close physical contact with the suspected or confirmed case of Nipah during the illness including during transportation • Has had direct close contact with the (deceased) suspected or confirmed case of Nipah at a funeral or during burial preparation rituals • Has touched the blood or body fluids (saliva, urine, vomitus etc.) of a suspected or confirmed case of Nipah during their illness • Has touched the clothes or linens of a suspected or confirmed case of Nipah

∗ In both suspected and probable cases for Nipah encephalitis, the patient might present with respiratory features with or without encephalitis. The respiratory features are (i) illness <7 days duration (ii) acute onset of fever (iii) severe shortness of breath, cough and (iv) chest radiograph showing diffuse infiltrates.

∗∗ The contact cases should be followed up for appearance of symptoms of Nipah infection for the longest incubation period (i.e., 21 days).

^1^ Definitions as described in the National Guideline for Management, Prevention and Control of Nipah Virus Infection including Encephalitis, Directorate General of Health Services, Ministry of Health & Family Welfare, Government of the People's Republic of Bangladesh; with technical support from WHO Bangladesh Office. Refer to Ref. [18].

^2^ Definition as described by the National Centre for Disease Control, Directorate General of Health Services, Ministry of Health and Family Welfare, Government of India. Refer to Ref. [19].

Alt-text: Box 1

Non-standard definitions compromise comparability across outbreaks and jurisdictions, distort denominators used to estimate severity (including case fatality), and can drive both under-ascertainment and false signals. Such ambiguity, originating from unclear or inconsistent reporting guidance, limits the reliability of pooled analyses and hampers the design of international multi-site diagnostic and therapeutic studies. This challenge is visible even in published “*total case*” estimates: counts of confirmed human Nipah cases differ by source and inclusion criteria. For example, a May 2024 hybrid surveillance-style compilation reported 754 confirmed cases globally, whereas a later August 2025 literature-derived synthesis identified only 717 disease cases, a discrepancy plausibly explained by differences in data sources, definitions applied, and de-duplication approaches used by the respective teams [22,23].

## 4 Choosing to catch early

Though numerically small, Nipah outbreaks are operationally high stakes, characterised by clinical heterogeneity, rapid deterioration in severe disease, and the potential for nosocomial amplification. Early signals therefore need to be interpretable across settings and jurisdictions. This matters because outbreak operations depend heavily on suspected and probable case definitions to determine who is sampled, isolated, traced, and followed; especially early, when laboratory capacity and healthcare systems may already be strained. In such contexts, non-uniform definitions can compound operational uncertainty and delay coordinated action. Accordingly, we propose the following near-term actions to strengthen readiness:

• **Develop minimum, tiered surveillance definitions:** WHO, in partnership with affected countries and other stakeholders, should publish minimum tiered case definitions (clinical-only, epidemiologically linked, contact tracing), explicitly designed for One Health (human–animal–environment) view and adaptable to differing resource levels.
• **Standardise a small core dataset for every reported case:** Alongside tiered definitions, we recommend a short, mandatory minimum reporting set comprising age/sex, geography, exposure category, clinical syndrome, date of onset, specimen type, test used, and clinical outcome, to enable rapid cross-jurisdiction comparability without constraining local operational protocols. This is particularly important since symptomatology maybe broader than typically assumed. A literature-derived synthesis found frequent gastrointestinal symptoms (diarrhoea, abdominal pain, hypersalivation, vomiting) alongside musculoskeletal symptoms (myalgia and arthralgia) [23]. Capturing these systematically reduces reliance on a narrow “systemic–respiratory–encephalopathic” template and supports earlier, more consistent detection across settings.
• **Harmonise contact definitions and follow-up windows:** We recommend adopting common terminology for contacts, clusters, and follow-up periods, including clear thresholds for “high-risk contact” and minimum expectations for tracing and symptom monitoring – particularly for healthcare-associated exposures.
• **Require transparent reporting in the scientific literature:** Journals, reviewers, and authors should treat explicit description of case definitions, diagnostic assays, specimen types, and de-duplication methods as essential reporting. This is further justified by the observation that only 44% (25/56) of eligible human case studies reported a clinical case definition, with marked variation among those that did [23]. We recommend that Nipah outbreak reports should be STrengthening the Reporting of OBservational studies in Epidemiology (STROBE) compatible *plus* include a dedicated outbreak information appendix.
• **Extend preparedness and awareness to high-contact, aerosol-generating clinical settings:** Given that early symptoms can be non-specific and transmission risk is concentrated in close-contact care, we recommend that preparedness guidance should incorporate Nipah-specific triage and infection-control pathways for healthcare and dental settings, including for aerosol-generating procedures (such as bronchoscopy, non-invasive ventilation, intubation, nebulizer treatment) [24], with deferral and referral triggers aligned to the surveillance definitions above.

Ultimately, we hope that by shining light on this gap, “*catching Nipah early*” will not be only about better diagnostics, treatment modalities, and preventive border measures; it will also be about ensuring that signals represent the same case-mix across clinics, laboratories, and countries. A shared surveillance language will turn scattered alerts into comparable evidence. Without it, we will risk repeatedly catching up later.

## Acknowledgement

None to declare.

## CRediT authorship contribution statement

**Hester Lacey:** Data curation, Formal analysis, Investigation, Validation, Writing – original draft, Writing – review & editing. **Shivani Jain:** Investigation, Methodology, Project administration, Resources, Supervision, Validation, Writing – review & editing. **Aigars Reinis:** Formal analysis, Funding acquisition, Investigation, Project administration, Resources, Supervision, Validation, Writing – review & editing. **Nityanand Jain:** Conceptualization, Data curation, Funding acquisition, Formal analysis, Investigation, Methodology, Project administration, Resources, Software, Supervision, Validation, Visualization, Writing – original draft, Writing – review & editing.

## Ethical approval

Not applicable.

## Funding

None to declare.

## Declaration of competing interest

The authors declare that they have no known competing financial interests or personal relationships that could have appeared to influence the work reported in this paper.

## References

[1] World Health Organization (WHO) (2026). https://www.who.int/emergencies/disease-outbreak-news/item/2026-DON593.

[2] National Centre for Disease Control (NCDC) (2026). NCDC, directorate general of health services.

[3] World Health Organization (WHO) (2026). https://www.who.int/emergencies/disease-outbreak-news/item/2026-DON594.

[4] Andres G. (Feb 22, 2026). Singapore to ease measures against Nipah virus from Feb 23; no cases reported to date. The Strait Times. https://www.straitstimes.com/singapore/spore-to-ease-measures-against-nipah-virus-from-feb-23-no-cases-reported-to-date.

[5] Singh R.K., Dhama K., Chakraborty S., Tiwari R., Natesan S., Khandia R., Munjal A., Vora K.S., Latheef S.K., Karthik K., Singh Malik Y., Singh R., Chaicumpa W., Mourya D.T. (2019 Dec). Nipah virus: epidemiology, pathology, immunobiology and advances in diagnosis, vaccine designing and control strategies - a comprehensive review. Vet Q.

[6] Larsen B.B., McMahon T., Brown J.T., Wang Z., Radford C.E., Crowe J.E., Veesler D., Bloom J.D. (2025 May 1). Functional and antigenic landscape of the Nipah virus receptor-binding protein. Cell.

[7] Liu Q., Stone J.A., Bradel-Tretheway B., Dabundo J., Benavides Montano J.A., Santos-Montanez J., Biering S.B., Nicola A.V., Iorio R.M., Lu X., Aguilar H.C. (2013). Unraveling a three-step spatiotemporal mechanism of triggering of receptor-induced Nipah virus fusion and cell entry. PLoS Pathog.

[8] Moore K.A., Mehr A.J., Ostrowsky J.T., Ulrich A.K., Moua N.M., Fay P.C., Hart P.J., Golding J.P. (2024 Nov). Measures to prevent and treat Nipah virus disease: research priorities for 2024-29. Lancet Infect Dis.

[9] Bruno L., Nappo M.A., Ferrari L., Di Lecce R., Guarnieri C., Cantoni A.M., Corradi A. (2022 Dec 31). Nipah virus disease: epidemiological, clinical, diagnostic and legislative aspects of this unpredictable emerging zoonosis. Animals (Basel).

[10] Hassan M.Z., Rojek A., Olliaro P., Horby P. (2025 Jan 3). Improving clinical care of patients in Nipah outbreaks: moving beyond 'compassionate use'. Lancet Reg Health Southeast Asia.

[11] Chua K.B. (2003 Apr). Nipah virus outbreak in Malaysia. J Clin Virol.

[12] Epstein J.H., Anthony S.J., Islam A., Kilpatrick A.M., Ali Khan S., Balkey M.D., Ross N., Smith I. (2020 Nov 17). Nipah virus dynamics in bats and implications for spillover to humans. Proc Natl Acad Sci U S A.

[13] Gaudino M., Aurine N., Dumont C., Fouret J., Ferren M., Mathieu C., Reynard O., Volchkov V.E., Legras-Lachuer C., Georges-Courbot M.C., Horvat B. (2020 Jan). High pathogenicity of Nipah virus from Pteropus lylei fruit Bats, Cambodia. Emerg Infect Dis.

[14] Breed A.C., Field H.E., Smith C.S., Edmonston J., Meers J. (2010 Jun). Bats without borders: long-distance movements and implications for disease risk management. EcoHealth.

[15] Hafeez M.H., Ajmal H., Nadeem A., Tabassum S., Akilimali A. (2025 Mar 5). Navigating Nipah virus: insights, challenges, and recommendations. New Microbes New Infect.

[16] Halpin K., Rota P. (2014 Aug). A review of Hendra virus and Nipah virus infections in man and other animals. Zoonoses - Infections Affecting Humans and Animals.

[17] Hsu V.P., Hossain M.J., Parashar U.D., Ali M.M., Ksiazek T.G., Kuzmin I., Niezgoda M., Rupprecht C., Bresee J., Breiman R.F. (2004 Dec). Nipah virus encephalitis reemergence, Bangladesh. Emerg Infect Dis.

[18] Directorate General of Health Services (December 2011). Technical support from WHO Bangladesh Country Office.

[19] National Centre for Disease Control (2026). Directorate general of health services.

[20] Center for Disease Control, National Institute of Health (October 2023). Advisory on Nipah virus infection (F.1-22/Advisory/FEDSD/2022). Ministry of national health services regulations and coordination, government of Pakistan. https://nih.org.pk/wp-content/uploads/2023/10/Nipah-Virus-Advisory-NIH.pdf.

[21] National Institute for Communicable Diseases (NICD) (September 2023). Nipah virus (NiV) disease preparedness: update for physicians, accident & emergency practitioners and laboratorians. Outbreak Response Unit, Division of Public Health Surveillance and Response and Centre for Emerging Zoonotic & Parasitic Diseases and Centre for Respiratory Diseases and Meningitis.

[22] Khan S., Akbar S.M.F., Mahtab M.A., Uddin M.N., Rashid M.M., Yahiro T., Hashimoto T., Kimitsuki K., Nishizono A. (2024 Aug 26). Twenty-five years of Nipah outbreaks in Southeast Asia: a persistent threat to global health. IJID Reg.

[23] Hassan M.Z., Ibrahim S.K., Harriss E., Horby P., Olliaro P., Rojek A. (2026 Jan). Interpreting the natural history and pathogenesis of Nipah virus disease through clinical data, to inform clinical trial design: a systematic review. Lancet Microbe.

[24] Judson S.D., Munster V.J. (2019 Oct 12). Nosocomial transmission of emerging viruses via aerosol-generating medical procedures. Viruses.

